# Supplementary material for: Subgrouping patients with ischemic heart disease by means of the Markov cluster algorithm
Source: Commun Med (Lond). 2025 Aug 26;5:372. doi: 10.1038/s43856-025-01077-1 (PMC12381225; doi:10.1038/s43856-025-01077-1)
Supplement: Supplementary file 2 — Supplementary Information [file 43856_2025_1077_MOESM2_ESM.pdf]

**Supplementary Information**

**Manuscript title:** Subgrouping patients with ischemic heart disease by means of the Markov Cluster algorithm

**Authors:** Haue AD, Holm PC, et al.

**Supplementary Figure 1: Classification of new ischemic events.**

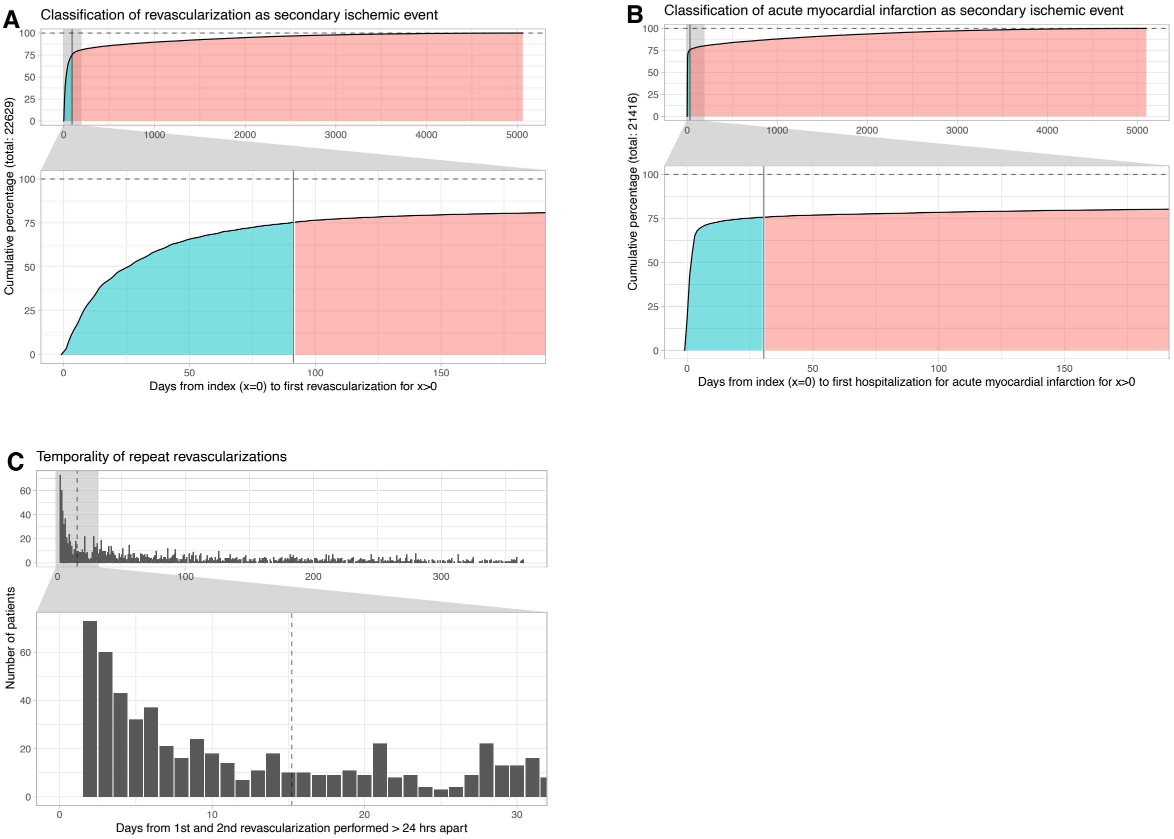


a: Time from index to first revascularization vs. percentage of patients revascularized. Blue corresponds to events related to establishment of IHD. Red corresponds to events considered new ischemic events. b: Time from index to first hospitalization for acute myocardial infarction vs. percentage of patients hospitalized. Blue corresponds to events related to index. Red corresponds to events considered new ischemic events. c: Distribution of days between revascularization for patients subjected to >1 performed >24 hours apart. Revascularizations performed <2 weeks apart were analyzed as a single event performed at date of the earliest revascularization. Marked by dashed line. IHD: Ischemic heart disease.

**Supplementary Figure 2: Selection of number of components.**

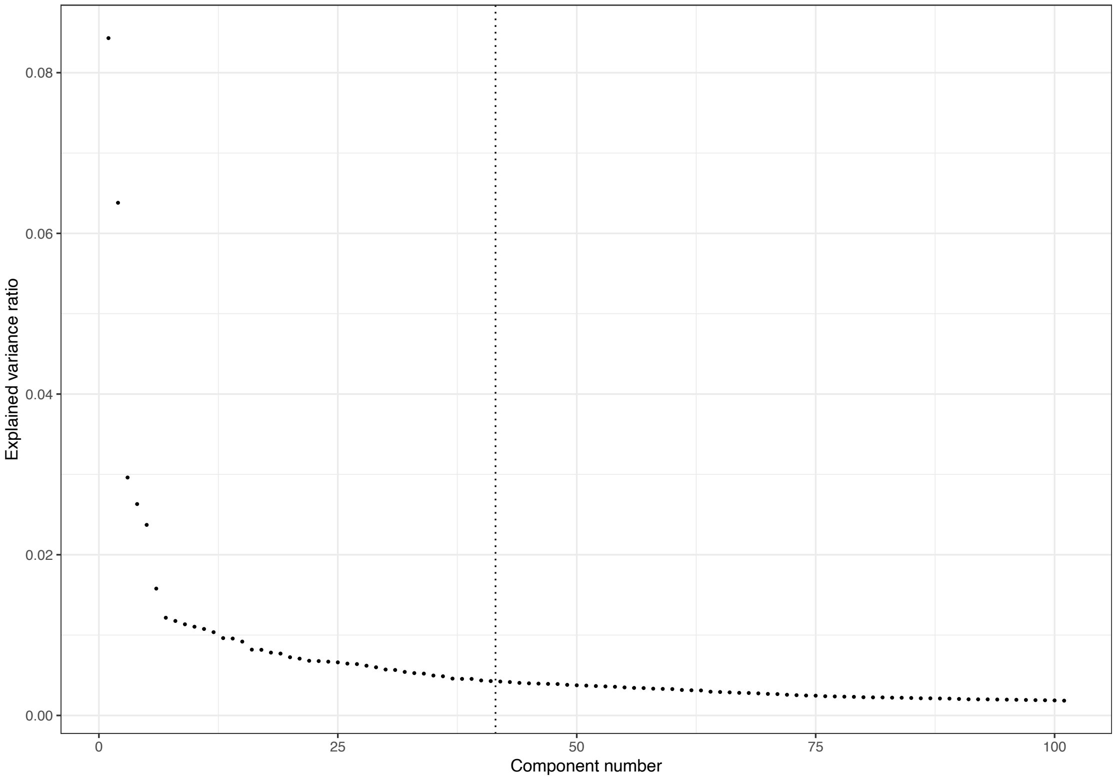


X-axis: Component number ranked by explained variance ratio. Y-axis: Explained variance ratio. Dashed horizontal line indicates the cutoff.

**Supplementary Figure 3: Limiting edge-density and average node degree in sex-specific similarity networks**.

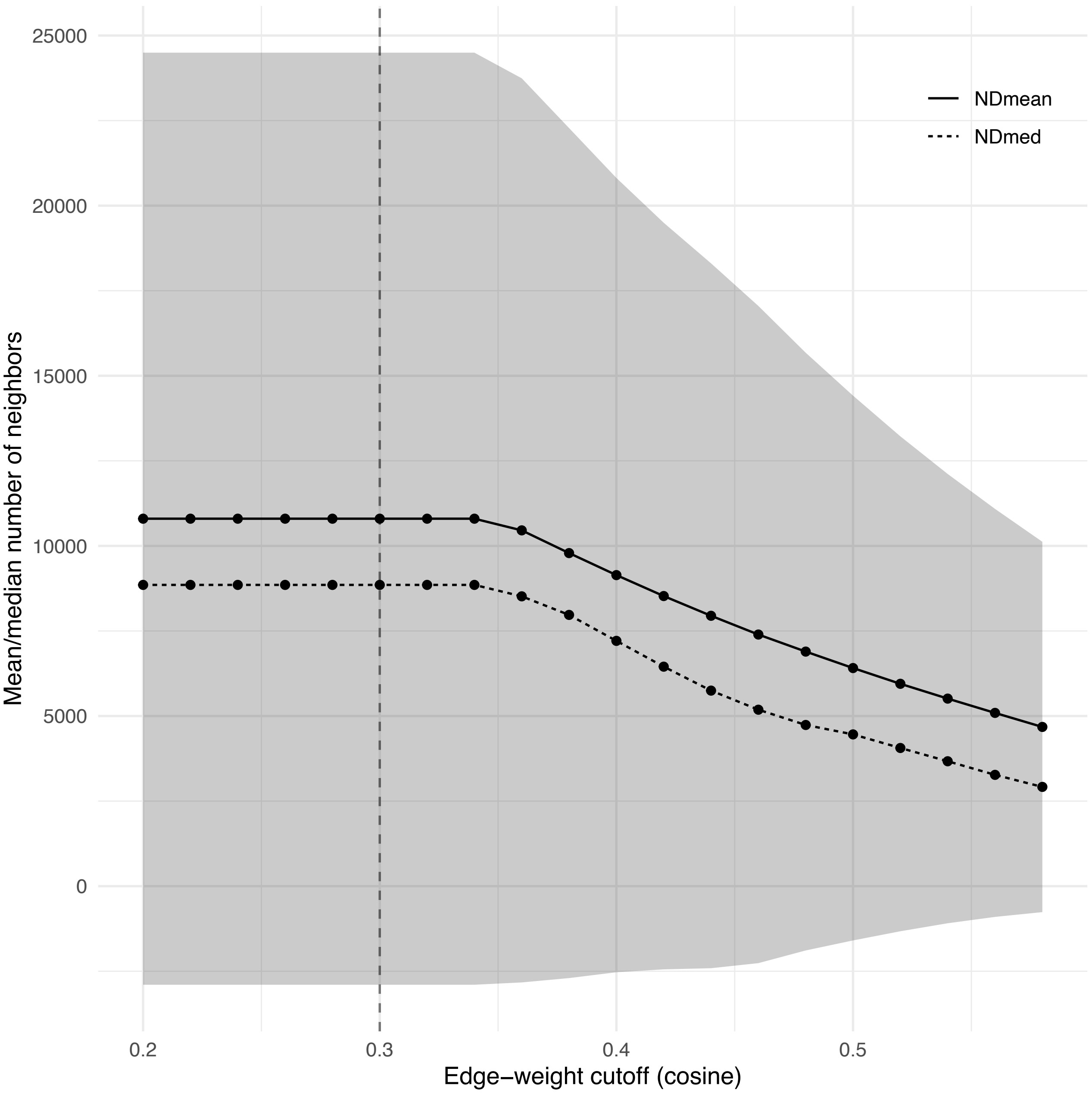


Mean/median number of neighbors against edge-weight cutoff in the patient similarity network. Only edges with a weight higher than 0.3 (as indicated by the vertical, dashed bar) were retained.

**Supplementary Figure 4: Results of robustness analysis by permutation.**

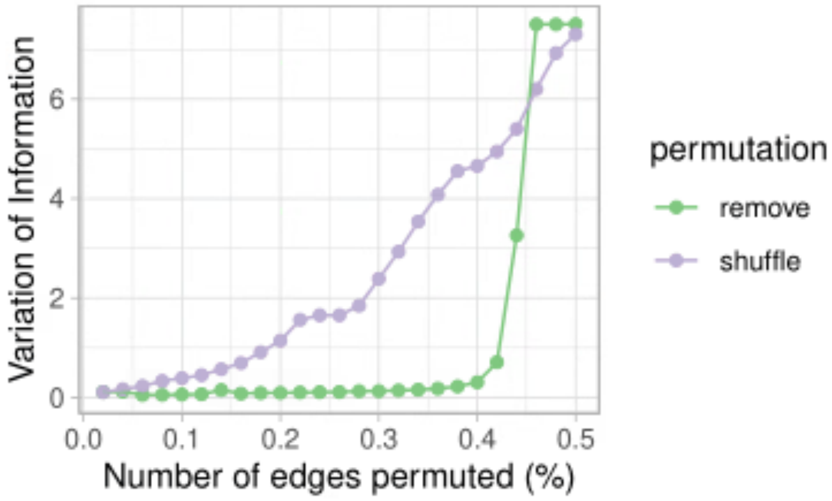


X-axis: Percentage of altered edges (deleted or removed). Y-axis: Variation of information measure compared to the reference graph. Legend: Type of alteration, with 10 mutations of the reference graph for each type.

**Supplementary Figure 5: Impact on network when diagnoses were excluded.**

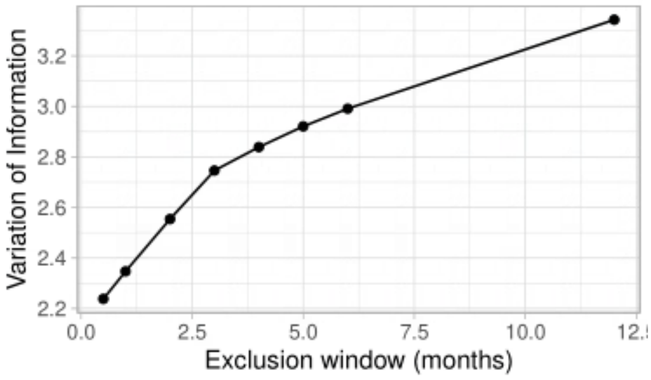


X-axis: Exclusion window in months. Y-axis: Variation of information.

**Supplementary Figure 6: Quantitative assessment of cluster robustness.**

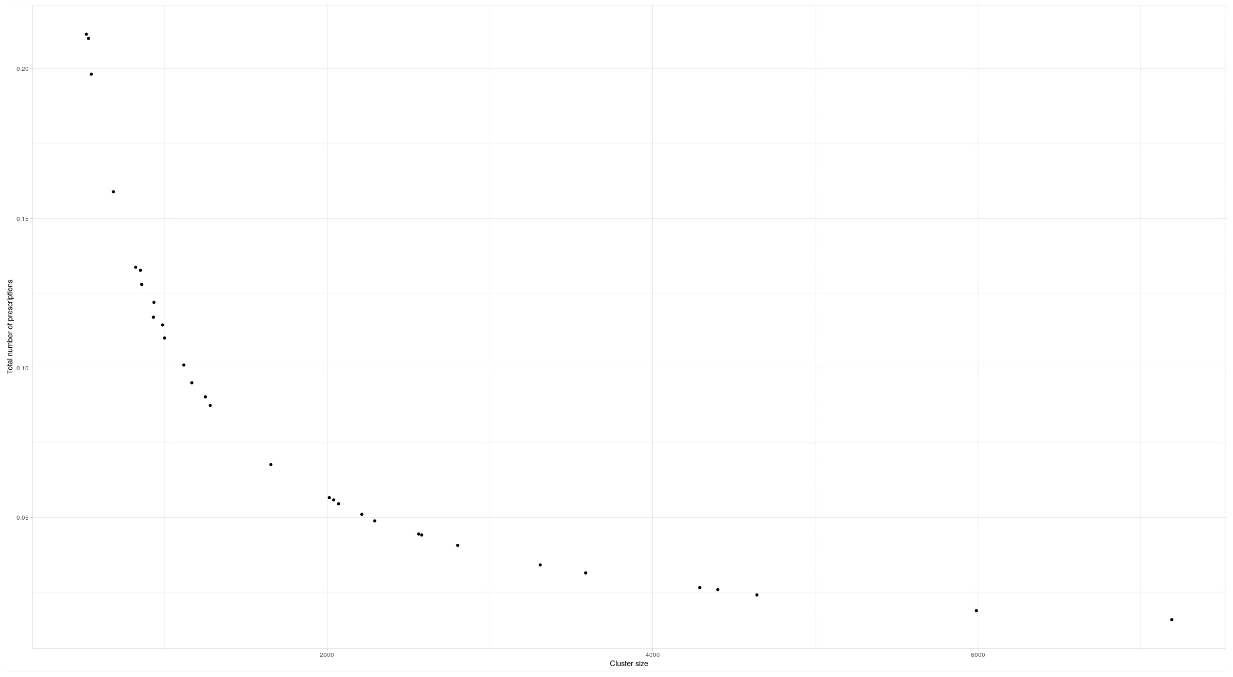


X-axis: Cluster size. Y-axis: Total number of redeemed prescriptions two years prior to index. One dot per cluster.

**Supplementary Figure 7: Risk of new ischemic events, non-IHD causes, and all-cause mortality stratified by cluster.**

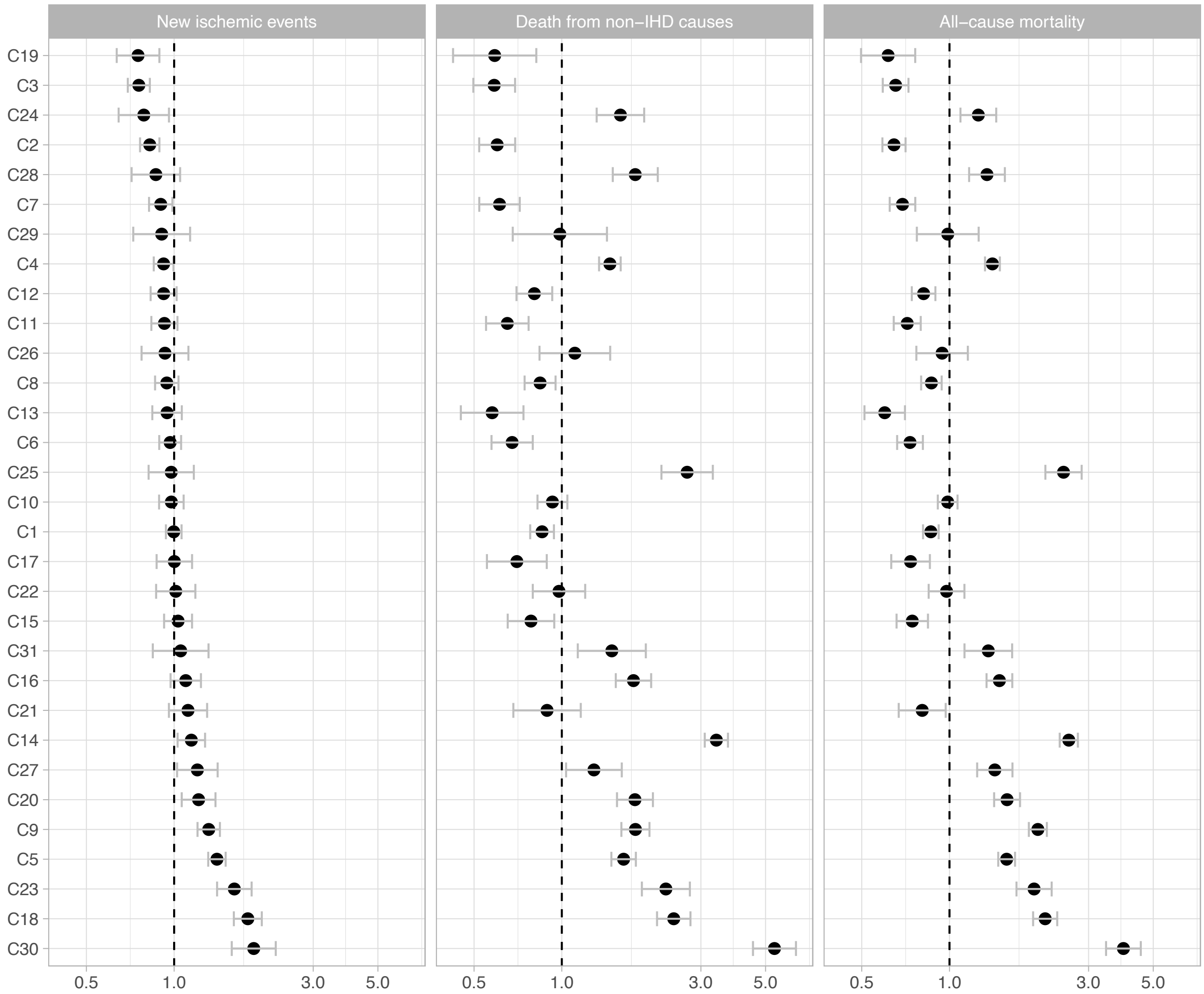


Forest plots where clusters are shown against HR for new ischemic events (left) and death from non-IHD causes (right). X-axis: HR for a single cluster relative to mean HR of the 30 other clusters. Y-axis: Clusters arranged by risk of new ischemic events, increasing risk from top to bottom. Error bars indicate adjusted 95% confidence intervals. IHD: Ischemic heart disease. HR: Hazzard ratio.

**Supplementary Figure 8: Heatmap of clusters based on laboratory profiles.**

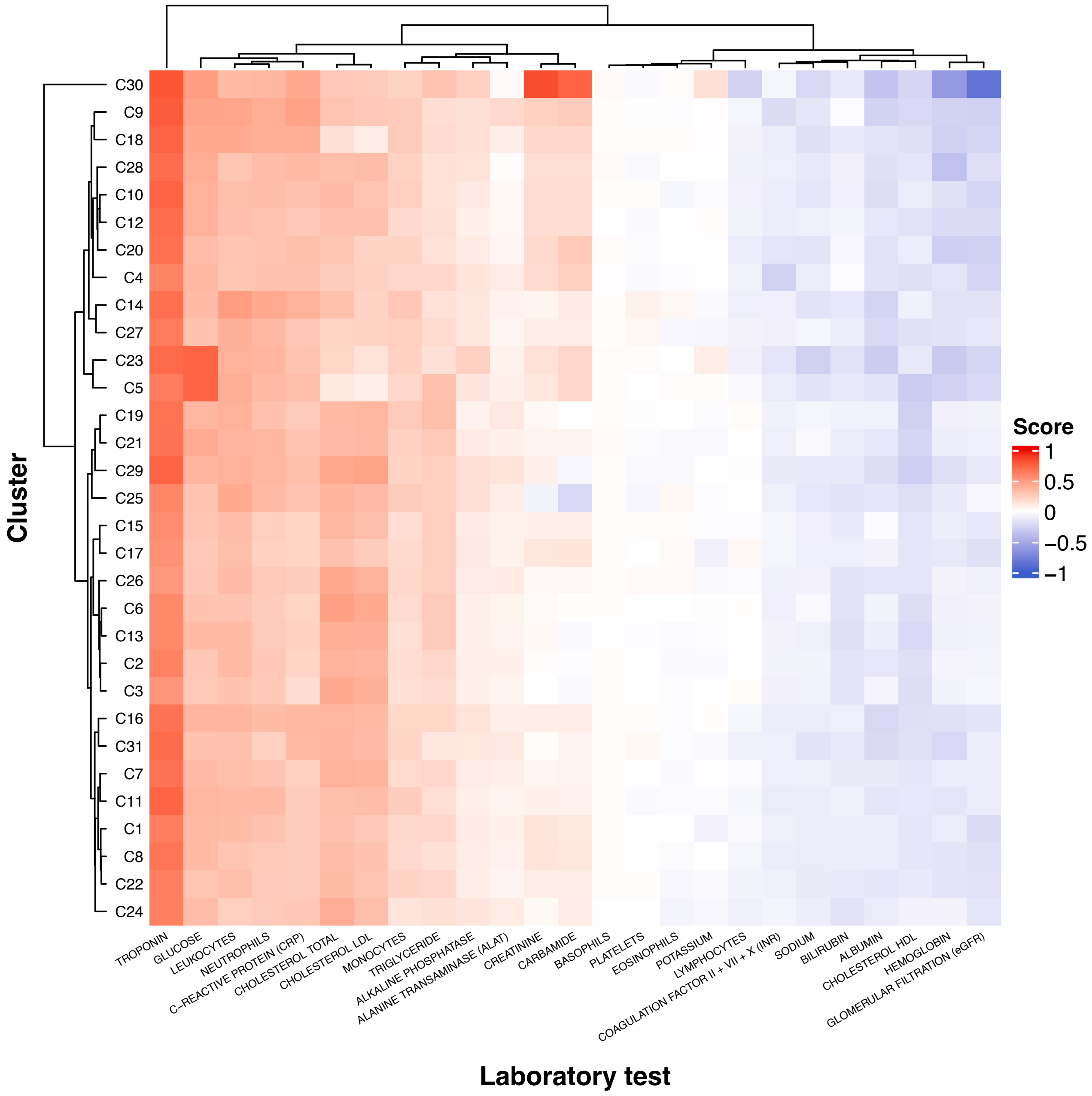


Summary of results from the phenotypic characterization of clusters based on laboratory data. *Score* refers to the mean summarized values per cluster, where values were assigned based on the results of the laboratory test per patient. Values of -1, 0, and 1, indicates below, within or above reference range, respectively. For details, see Methods. X-axis: Laboratory test. Y-axis: Cluster.

**Supplementary Table 1: Eligible codes for inclusion and outcomes**

| ICD-10[1] chapter IX | | | Definition, level 3 |
|---|---|---|---|
| **Block** | **Level 3** | **Level 4** | |
| | I20 | I20.0*, I20.1, I20.8, I20.9 | Angina pectoris |
| | I21* | I21.0, I21.1, I21.2, I21.3, I21.4, I21.9 | Acute myocardial infarction |
| **R94** | I23 | I25.0, I25.1, I25.2, I25.23, I25.4, I25.5, I25.6, I25.8, I25.9 | Certain current complications following acute myocardial infarction |
| | I24 | I24.0, I24.1, I24.8, I24.9 | Certain current complications following acute myocardial infarction |
| | I25 | I25.0, I25.1, I25.2, I25.23, I25.4, I25.5, I25.6, I25.8, I25.9 | Chronic ischemic heart disease |
| **Nomesco[2] code** | | **Procedure** | |
| FNA* | | Connection to coronary artery from internal mammary artery | |
| FNB* | | Connection to coronary artery from gastroepiploic artery | |
| FNC* | | Aorto-coronary venous bypass | |
| FND* | | Aorto-coronary bypass using prosthetic graft | |
| FNE* | | Coronary bypass using free arterial graft | |
| FNF* | | Coronary thrombendarterectomy | |
| FNG* | | Expansion and recanalisation of coronary artery | |
| **SKS[3] code** | | **Procedure** | |
| UXAC85[A-D] | | Coronary arteriography | |
| UXCC00A | | Coronary computed tomography angiography | |
| **SHAK[4] code** | | **Hospital** | |
| 1301 | | Rigshospitalet | |
| 1309 | | Bispebjerg og Frederiksberg Hospitaler | |
| 1330 | | Amager og Hvidovre Hospital | |
| 1351 | | Amager Hospital | |
| 1401 | | Frederiksberg Hospital | |
| 1501 | | Gentofte Hospital | |
| 1502 | | Glostrup Hospital | |
| 1516 | | Herlev og Gentofte Hospital | |
| 2000 | | Hospitalerne i Nordsjælland | |
| 2501 | | Amtssygehuset i Roskilde | |
| 3800 | | Region Sjællands Sygehusvæsen | |
| 4001 | | Bornholms Hospital | |

[1] ICD-10 = WHO International classification of diseases and health related problems 10th edition. Danish version where code types A, B and G included in our definition of primary and secondary codes.

[2] NOMESCO = Nordic Medico-Statistical Committee

[3] SKS = Sundhedsstyrelses klassifikationsstystem [Danish]

[4] SHAK = Sygehus- og afdelingsklassifikation [Danish]

[*] Included in the composite outcome new ischemic events. For ICD-10 codes only code types A (primary) and in-hospital patients.

**Supplementary Table 2: Laboratory codes included in assessment of data quality and completeness**

| Blood analyte | NPU codes and local systems |
|---|---|
| Sodium | NPU03429, GEN00992, NPU03796, POC00022, 240, POC00021, POC00023, GEN00990 |
| Potassium | NPU03230, GEN00995, POC00019, POC00018, POC00020, GEN00993 |
| Hemoglobin | NPU02319, GEN00989, NPU02321, NPU02320, NPU02322, NPU17007, POC00013, NPU04208, NPU01393, POC00012, POC00014, NPU29057, GEN00987 |
| Creatinine / EGFR | NPU04998, NPU03918, NPU09102, NPU19661, NPU14048, NPU03800, HLL00037, DNK35131, POC00109, RHB00941, NPU28842 |

**Supplementary Table 3: Demographics for patients that did not cluster or were in clusters of size < 500**

| **Cohort demographics** | **Total** | **Males** | **Females** |
|---|---|---|---|
| Number of patients | 5,113 | 3,878 | 1,235 |
| Mean age at index (SD) | 60.7 | 60.0 | 63.0 |
| **Outcomes, number of cases** | **Total** | **Males** | **Females** |
| New ischemic events | 995 | 780 | 175 |
| Death from non-IHD causes | 352 | 274 | 78 |
| Censored | 3,624 | 2,707 | 917 |
| **Outcomes, time to event** | **Mean time to event in years (SD)** | | |
| | **Total** | **Males** | **Females** |
| New ischemic events | 1.55 (1.41) | 1.59 (1.43) | 1.39 (1.32) |
| Death from non-IHD causes | 2.25 (1.50) | 2.18 (1.47) | 2.5 (1.49) |
| Censored | 4.54 (0.95) | 4.52 (0.96) | 2.47 (1.49) |
| Total | 4.02 (1.52) | 3.98 (1.54) | 4.17 (1.44) |

**Supplementary Table 4: Chi-squared test for distribution laboratory values in clusters**

| Component | P-val. | Adj. P-val. |
|---|---|---|
| Alanine transaminase (ALAT) | 4.78 e-22 | 1.15e-20 |
| Albumin | 4.81e-22 | 1.15e-20 |
| Alkaline phosphatase | 2.01e-22 | 4.82e-21 |
| Bilirubin | 1.09e-13 | 2.60e-12 |
| C-reactive protein (CRP) | 1.65e-96 | 3.95e-95 |
| Carbamide | 5.49-e200 | 1.32e-198 |
| Cholesterol HDL | 1.99e-66 | 4.77e-65 |
| Cholesterol LDL | 4.86e-53 | 1.17e-51 |
| Cholesterol total | 2.64e-58 | 6.34 e-57 |
| Coagulation factor II + VII + X | 7.96e-280 | 1.91e-278 |
| Creatinine | 9.28e-302 | 2.23e-300 |
| Eosinophils | 4.43e-6 | 1.06e-4 |
| Estimated glomerular filtration rate (eGFR) | 0 | 0 |
| Glucose | 0 | 0 |
| Hemoglobin | 2.77e-218 | 6.65e-217 |
| Leukocytes | 1.42e-39 | 3.41e-38 |
| Lymphocytes | 1.54e-17 | 3.69e-16 |
| Monocytes | 1.06e-11 | 2.55e-10 |
| Neutrophils | 5.69e-20 | 1.36e-18 |
| Platelets | 2.39e-23 | 5.73e-22 |
| Potassium | 9.03e-32 | 2.17e-30 |
| Sodium | 2.24e-74 | 5.38e-74 |
| Triglyceride | 2.10e-60 | 5.04 e-59 |
| Troponin | 7.10e-73 | 1.70e-71 |

## Supplementary Table 5: Traits with significantly different PGS distributions in clusters*

| Cluster | n | trait | effect | effect size | FDR |
|---|---|---|---|---|---|
| C1 | 2,025 | Systolic Blood Pressure | + | 0.20 | <0.0005 |
| | | Diastolic Blood Pressure | + | 0.16 | <0.0005 |
| | | Total Cholesterol | - | -0.08 | 0.026 |
| C4 | 1,532 | Atrial Fibrillation | + | 0.57 | <0.0005 |
| | | Heart Failure | + | 0.08 | 0.031 |
| | | Coronary Artery Disease | - | -0.12 | 0.001 |
| | | T2D (BMI-adj.) | - | -0.11 | 0.001 |
| | | Acute Myocardial Infarction | - | -0.08 | 0.031 |
| | | Triglyceride | - | -0.08 | 0.044 |
| | | Total Cholesterol | - | -0.08 | 0.046 |
| C5 | 1,136 | T2D (BMI-adj.) | + | 0.55 | <0.0005 |
| | | NAFLD | + | 0.11 | 0.021 |
| C6 | 860 | Total Cholesterol | + | 0.21 | <0.0005 |
| | | Triglyceride | + | 0.20 | <0.0005 |
| | | LDL Cholesterol | + | 0.15 | 0.001 |
| | | Coronary Artery Disease | + | 0.15 | 0.001 |
| | | Diastolic Blood Pressure | - | -0.13 | 0.015 |
| | | Systolic Blood Pressure | - | -0.11 | 0.040 |
| C8 | 817 | Systolic Blood Pressure | - | -0.16 | 0.001 |
| | | Stroke | - | -0.12 | 0.023 |
| | | Coronary Artery Disease | - | -0.12 | 0.028 |
| | | Diastolic Blood Pressure | - | -0.11 | 0.031 |
| | | LDL Cholesterol | - | -0.10 | 0.047 |
| C10 | 744 | Coronary Artery Disease | - | -0.13 | 0.017 |
| | | Acute Myocardial Infarction | - | -0.13 | 0.021 |
| C11 | 718 | Stroke | - | -0.11 | 0.040 |
| | | Heart Failure | - | -0.11 | 0.040 |
| C12 | 649 | Coronary Artery Disease | - | -0.14 | 0.013 |
| | | Acute Myocardial Infarction | - | -0.12 | 0.033 |
| C13 | 606 | Diastolic Blood Pressure | - | -0.12 | 0.040 |
| C15 | 588 | LDL Cholesterol | + | 0.14 | 0.020 |
| | | Total Cholesterol | + | 0.14 | 0.026 |
| C17 | 348 | Systolic Blood Pressure | + | 0.16 | 0.040 |
| C18 | 481 | T2D (BMI-adj.) | + | 0.15 | 0.023 |
| | | Acute Myocardial Infarction | + | 0.15 | 0.028 |
| | | Atrial Fibrillation | - | -0.13 | 0.049 |
| C23 | 290 | T2D (BMI-adj.) | + | 0.27 | 0.001 |
| C25 | 297 | Coronary Artery Disease | + | 0.24 | 0.002 |
| | | Acute Myocardial Infarction | + | 0.18 | 0.032 |
| | | Heart Failure | + | 0.18 | 0.031 |
| C27 | 231 | Stroke | + | 0.24 | 0.015 |

*For each cluster we tested if the PGS distribution in that specific cluster differs from the PGS distribution in all other clusters using the Wilcoxon rank-sum test. A positive effect size for a specific trait means individuals in the cluster on average have *higher* PGS values for that trait than individuals in the other clusters combined, and vice versa. Effect sizes reflect the magnitude of the difference between PGS distributions. PGS = polygenic score, n = number of individuals in the cluster, FDR = false discovery rate, BMI-adj. = adjusted for Body Mass Index, NAFLD = non-alcoholic fatty liver disease, LDL = low density lipoprotein.
