# Supplementary material for: Subgrouping patients with ischemic heart disease by means of the Markov cluster algorithm
Source: Commun Med (Lond). 2025 Aug 26;5:372. doi: 10.1038/s43856-025-01077-1 (PMC12381225; doi:10.1038/s43856-025-01077-1)
Supplement: Supplementary file 3 — Description of Additional Supplementary files [file 43856_2025_1077_MOESM3_ESM.pdf]

Description of Additional Supplementary files

File name: Supplementary Data 1

Description: Comparison of mean age at index in 31 cluster using Tukey's HSD.

File name: Supplementary Data 2

Description: Source data for Figure 4 575 is available in Supplementary Data 2.

File name: Supplementary Data 3

Description: The source data for Figure 3 is available in Supplementary Data 3.
